# Identification of viral SIM-SUMO2-interaction inhibitors for treating primary effusion lymphoma

**DOI:** 10.1371/journal.ppat.1008174

**Published:** 2019-12-12

**Authors:** Ling Ding, Qing Zhu, Feng Zhou, Hongsheng Tan, Wenjia Xu, Chengling Pan, Caixia Zhu, Yuyan Wang, Hong Zhang, Wenwei Fu, Zhikang Qian, Zhenghong Yuan, Hongxi Xu, Fang Wei, Qiliang Cai

**Affiliations:** 1 MOE& NHC&CAMS Key Laboratory of Medical Molecular Virology, School of Basic Medical Science, Shanghai Medical College, Fudan University, Shanghai, P. R. China; 2 ShengYushou Center of Cell Biology and Immunology, School of Life Sciences and Biotechnology, Shanghai Jiao Tong University, Shanghai, P. R. China; 3 Baoji Affiliated Hospital of Xi’an Medical University, Baoji & MOE Key Laboratory of Western Resources and Modern Biotechnology, College of Life Sciences, Northwest University, Xi’an, Shaanxi, China; 4 School of Pharmacy, Shanghai University of Traditional Chinese Medicine & Engineering Research Center of Shanghai Colleges for TCM New Drug Discovery, Shanghai, China; 5 Unit of Herpesvirus and Molecular Virology, Key Laboratory of Molecular Virology & Immunology, Institut Pasteur of Shanghai, Chinese Academy of Sciences, University of the Chinese Academy of Sciences, Shanghai, P. R. China; 6 Beijing Computing Center, Beijing Academy of Science and Technology & Beijing Beike Deyuan Bio-Pharm Technology Company, Beijing, P. R. China; 7 Expert Workstation, Baoji Central Hospital, Baoji, P. R. China; University of Southern California, UNITED STATES

## Abstract

Primary effusion lymphoma (PEL) is an aggressive B-cell malignancy without effective treatment, and caused by the infection of Kaposi’s sarcoma-associated herpesvirus (KSHV), predominantly in its latent form. Previously we showed that the SUMO2-interacting motif within the viral latency-associated nuclear antigen (LANA^SIM^) is essential for establishment and maintenance of KSHV latency. Here, we developed a luciferase based live-cell reporter system to screen inhibitors selectively targeting the interaction between LANA^SIM^ and SUMO2. Cambogin, a bioactive natural product isolated from the *Garcinia genus* (a traditional herbal medicine used for cancer treatment), was obtained from the reporter system screening to efficiently inhibit the association of SUMO2 with LANA^SIM^, in turn reducing the viral episome DNA copy number for establishment and maintenance of KSHV latent infection at a low concentration (nM). Importantly, Cambogin treatments not only specifically inhibited proliferation of KSHV-latently infected cells *in vitro*, but also induced regression of PEL tumors in a xenograft mouse model. This study has identified Cambogin as a novel therapeutic agent for treating PEL as well as eliminating persistent infection of oncogenic herpesvirus.

## Author summary

Primary effusion lymphoma is a common AIDS-associated malignancy caused by infection with Kaposi’s sarcoma-associated herpesvirus (KSHV), and is currently absence of efficient and specific treatment. Natural product from herbal medicines is a major source of drug discovery for the treatment of a variety of diseases. In this study, the authors demonstrated that Cambogin, a polycyclic polyprenylated acylphloroglucinols (PPAPs) isolated from the branches of *Garcinia esculenta* (a tropical evergreen tree and traditional cancer treatment across Southern Asia), is a potent and effective inhibitor of KSHV-latently infected cells at a low concentration (nM) *in vitro* and *in vivo*, through targeting viral LANA^SIM^-SUMO2 interaction.

## Introduction

Kaposi’s sarcoma-associated herpesvirus (KSHV), also known as human herpesvirus 8 (HHV-8), is an oncogenic γ-herpesvirus that discovered by Chang and Moore over two decades ago [1]. It has been etiologically linked to several cancers in humans, such as Kaposi’s sarcoma (KS), primary effusion lymphoma (PEL), and certain types of multicentric Castleman’s disease (MCD) [2]. KSHV typically causes malignancies in individuals that are immunocompromised due to many factors including HIV infection or immunosuppressive drug therapies used for organ transplantation.

PEL is a B-cell lymphoma that develops in the pleural space and other body cavities, also referred to as body cavity lymphoma [3]. All PEL tumors are latently infected with KSHV, and up to 70% of PEL cases are co-infected with Epstein-Barr virus (EBV, highly homologous to KSHV). Although highly active antiretroviral therapies have dramatically reduced the incidence of PEL in HIV-infected patients, PEL remains the common AIDS-associated malignancy in both developed and developing countries. There is currently no efficient and specific treatment for PEL, the tumor is often resistant to conventional cytotoxic chemotherapy and most patients die within months [4–6]. Hence, new therapies for PEL are urgently needed.

Similar to the other herpesviruses, KSHV infection also exhibits a biphasic life cycle of latent and lytic replication, and can establish a life-long persistence in the host cell after primary infection [7]. The majority of KSHV exists in a latent form in tumor cells, while a small population undergoes spontaneous lytic replication. Upon reactivation from latency, most viral genes are expressed in an orderly fashion, leading to production of infectious virions. Since viral reactivation also results in host cell destruction *in vitro*, lytic induction of latent KSHV is considered to be a potential therapeutic option for treatment of KSHV-associated malignancies [8,9]. Many antiviral drugs, such as ganciclovir, valproic acid, bortezomib, prostratin, anthracycline and celecoxib, are based on the target of lytic replication [10–15]. However, despite the therapeutic potential of these compounds, their effectiveness and side effects in PEL treatment has yet to be further evaluated *in vivo*. Therefore, more effective therapeutic candidates for KSHV need to be identified.

The latency-associated nuclear antigen (LANA), a potent multifunctional protein encoded by open reading frame (ORF) 73, is critical for establishment and maintenance of KSHV latent infection [16]. LANA is highly expressed and readily detected in all infected cells [17], which is not only essential for establishing and maintaining successful latency [18], but also contributes to angiogenesis and cell proliferation through regulation of viral and cellular gene expression [19–22]. Because the SUMO2-interacting motif (SIM) within LANA plays a critical role in maintenance of the KSHV episome during cell passage and lytic gene silencing [23], the development of therapeutic strategies against KSHV-related cancer by using LANA^SIM^ as a target is of great interest.

For decades, natural products from herbal medicines have remained a major source of drug discovery for the treatment of a variety of diseases. In this study, we developed a luciferase based live-cell reporter system to quantitatively measure the intermolecular interaction of LANA^SIM^ with SUMO2. This system was used to screen natural product inhibitors isolated from different herbal medicines. Cambogin, a polycyclic polyprenylated acylphloroglucinols (PPAPs) isolated from the branches of *Garcinia esculenta* (a tropical evergreen tree and traditional cancer treatment across Southern Asia), was identified as a potent and effective inhibitor of KSHV latent and primary infection at a low concentration (nM), as it blocked LANA^SIM^-SUMO2 interaction. Moreover, Cambogin is an effective inhibitor of PEL cell growth *in vitro* and *in vivo*.

## Results

### Identification of natural product inhibitors of the interaction between LANA^SIM^ and SUMO2

**Fig 1:**
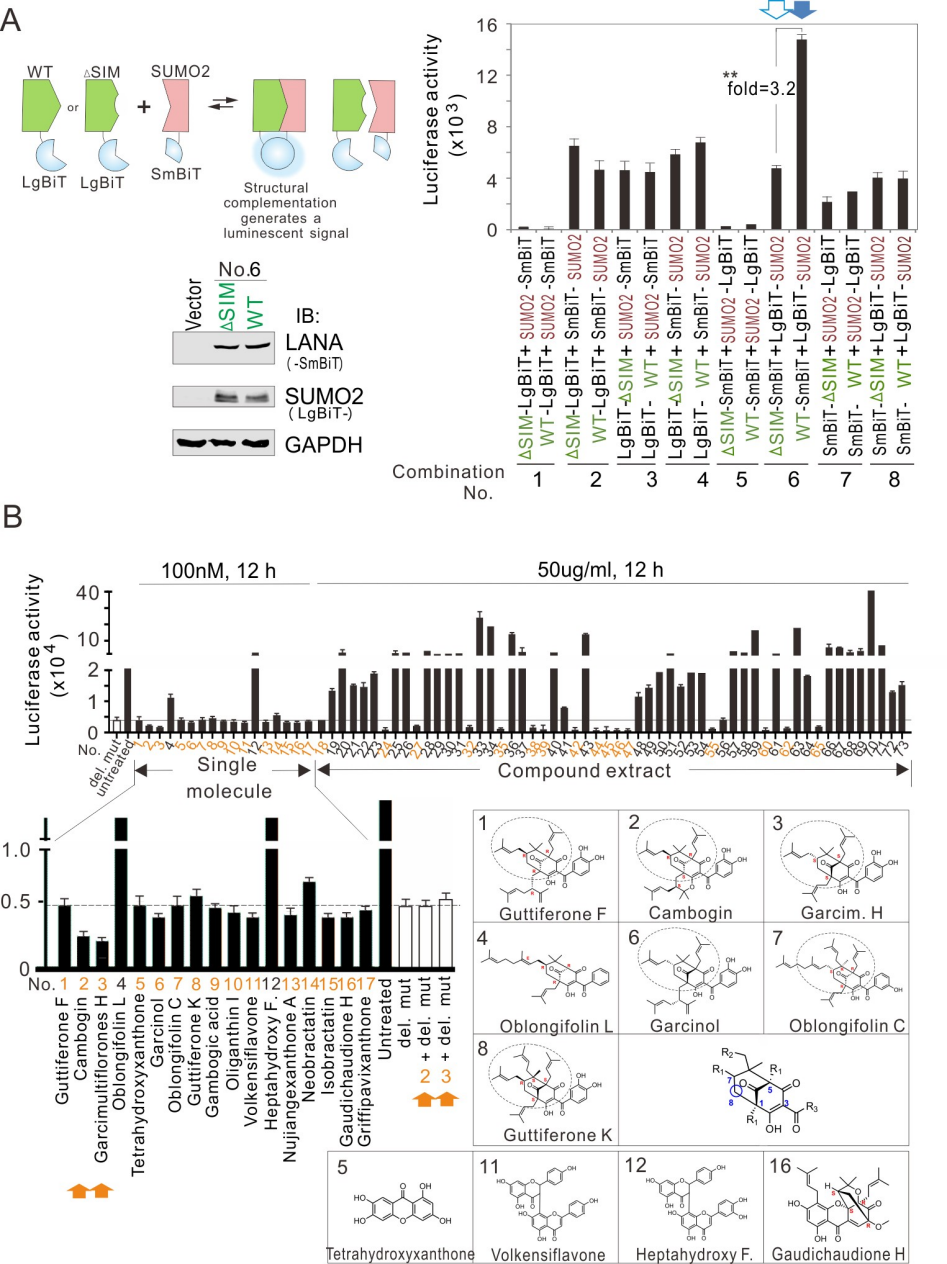
Identification of small molecules that block the intracellular interaction of viral SIM and SUMO2. (**A**) Establishment of a live-cell system for quantitatively measuring the intracellular interaction of viral SIM and SUMO2 using NanoLuc Binary Technology. HEK293 cells were individually transfected with different combinations of wild-type (WT) LANA [or its SIM-deleted (ΔSIM) mutant] and SUMO2 fusion with large or small fragments of BiT (LgBiT or SmBiT) for 48 h, incubated with Nano-GloR reagent, and luminescence was measured. Among the eight combinations, the luciferase activity of the WT LANA-SmBiT and LgBiT-SUMO2 combination (arrow) exhibited the highest level in three-independent experiments. The effect was dramatically abolished by deletion of the SIM motif. *Left panels*, the schematic for establishing the live-cell system to quantitatively measure the intracellular interaction of viral SIM and SUMO2. *Bottom panels*, the immunoblotting analysis of cell lysate from combination No. 6 transfection along with vector alone as a control. (**B**) Representative screening of 73 compounds from Chinese herbal medicines. HEK293 cells co-transfected with WT LANA-SmBiT and LgBiT-SUMO2 were untreated or treated with each compound for 12 h. The SIM-deleted mutant (ΔSIM) untreated or treated with compound No. 2 or No. 3 were used as a parallel control. *Bottom panels*, the chemical structures of representative compounds of PPAPs and Xanthones. Core skeleton of type-B PPAPs is highlighted. The details of each compound see supplementary **S1 Table**.

Previous studies showed that the SIM motif of LANA plays a critical role in the maintenance of the KSHV episome during cell passage and lytic gene silencing [23]. To explore drugs that may act against KSHV and related cancer cells through targeting LANA^SIM^, we developed a luciferase based live-cell reporter assay (containing two subunits of SmBiT and LgBiT) to quantitatively measure the interaction of LANA^SIM^ with SUMO2 via NanoLuc Binary Technology (Fig 1A). A total eight pairs of wild-type (WT) LANA, its SIM-deleted mutant (ΔSIM), and SUMO2 fused with either SmBiT or LgBiT on different terminals were individually constructed and tested in HEK293 cells for specific interaction of LANA with SUMO2 via the SIM domain (Fig 1A). The results showed that the combination of WT-SmBiT and LgBiT-SUMO2 presented the highest luminescence activity and showed the largest difference (~3.2 fold) between the WT and SIM-deleted mutant (ΔSIM-SmBiT) (Fig 1A, lane 6), making it suitable for inhibitor screening.

To identify inhibitors of the interaction of LANA^SIM^ and SUMO2, HEK293 cells were co-transfected with LANA WT-SmBiT and LgBiT-SUMO2, and then individually treated with 73 active compounds of natural products isolated from the Chinese traditional herbal medicines (which have been highly screened from thousands of compounds against cancer and deposited at SUTCM, supplemental S1 Table) at 50 μg/ml (a low-cytotoxicity concentration, and approximately 100 nmol/L(nM) based on molecular weight of selected single-molecule compound) concentration for 12 h, followed by luminescence assay. The results showed that 44.6% (25/56) of compound extracts presented dramatically inhibitory activities on the interaction of LANA^SIM^ and SUMO2 to some extent (Fig 1B). To further discover the specific inhibitors, 17 compounds from *Garcinia* plant that are well-characterized as single molecule compounds were used to test (Fig 1B, No.1 to 17). Interestingly, the compounds with similar core structures of type-B PPAPs (No. 1 to 3, and 6 to 8) or Xanthones (No. 5, 11, and 16) consistently presented higher inhibitory activities on the interaction of LANA^SIM^ and SUMO2, while both PPAP compound No.4 (Oblongifolin L) and Xanthones compound No.12 (Heptahydroxy F.) with a methyl or hydroxyl substituent totally abolished their inhibitory activities (Fig 1B, bottom panels) when compared to No. 3 (Garcimultiflorones H) and No. 11 (Volkensiflavone), respectively. These indicate that the core skeleton structure of both PPAP and Xanthones compounds present inhibitory activities in the interaction of LANA^SIM^ and SUMO2, and the activity varies due to the type and number of substituents on the specific position of core skeleton structure. Strikingly, among these inhibitors, No. 2 (Cambogin) and No. 3 (Garcimultiflorone H) consistently presented higher inhibitory activities on the interaction of WT LANA^SIM^ and SUMO2, but not in its SIM-deleted mutant group (Fig 1B).

### Cambogin blocked the association of LANA^SIM^ with SUMO2

**Fig 2:**
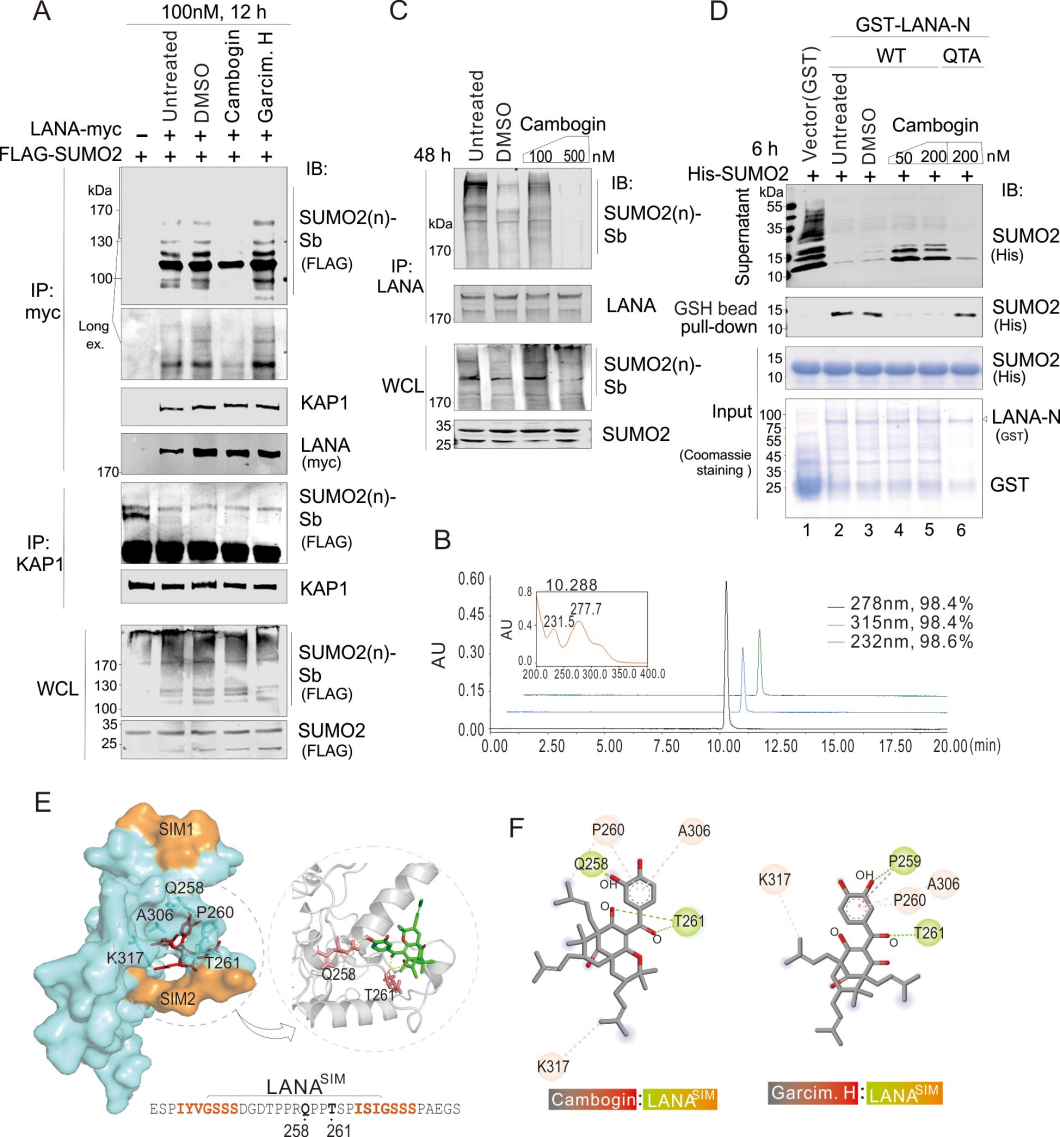
Cambogin specifically blocks the association of LANA^SIM^ with SUMO2. (**A**) Cambogin but not Garcimultiflorone dramatically blocked the intracellular interaction of exogenous LANA and SUMO2. Whole cell lysates (WCL) from HEK293 cells transfected with myc-tagged LANA and FLAG-tagged SUMO2 were individually treated with compound (100 nM final concentration), DMSO or untreated for 12 h at 24 h post-transfection, and subjected to immunoprecipitation (IP) and immunoblotting (IB) with the indicated antibodies. The LANA-interacting SUMO2 modified substrates [SUMO2(n)-sb] is shown in line. The position (>170 kDa) of SUMO2 modified substrates [SUMO2(n)-sb] from immunoprecipitated complex is enlarged. (**B**) UPLC chromatogram of Cambogin. Purity of Cambogin was shown on the right panel. (**C**) Cambogin efficiently inhibited the interaction of endogenous LANA and SUMO2 in PEL cells. Whole cell lysates (WCL) from BCP1 cells were individually treated with (100, 500 nM) Cambogin or DMSO for 48 h, and subjected to immunoprecipitation (IP) and immunoblotting (IB) with the indicated antibodies. The untreated group was used as a control. The LANA-interacting SUMO-2 modified substrates [SUMO2(n)-sb] is shown in line. (**D**) Cambogin efficiently blocked the interaction between LANA and SUMO2 *in vitro*. Equal protein amounts of purified His-SUMO2 were co-incubated with GST or GST-LANA-N (WT or QTA) fusion proteins *in vitro* for 12 h, followed by treatment with different dosages of Cambogin for another 6 h. Untreated and DMSO were used as control groups. Glutathione (GSH)-sepharose beads were used and results were analyzed by immunoblotting (IB) with the indicated antibodies. An aliquot of the purified His-SUMO2 fusion proteins was also applied directly to the gel (Input), and the GST fusion proteins on the blot were visualized using Coomassie staining (bottom panel). (**E**) Molecular Docking of LANA^SIM^ with Cambogin. The docking results show that the small molecule Cambogin received the active center of the LANA^SIM^ protein. The amino acid sequence of the LANA^SIM^ domain is shown at the bottom panel. The two subunits (SIM1 and SIM2) of LANA^SIM^ are highlighted in orange. Right panel, a three-dimensional view of the interactions between the LANA^SIM^ domain and Cambogin by Pymol software. The formation of hydrogen bonds between Cambogin and residues Gln-258 and Thr-261 of LANA^SIM^ is enlarged. (**F**) A two-dimensional view of the interaction between LANA^SIM^ and Cambogin shown by Discovery Studio software. The compound Cambogin also forms hydrophobic interactions with Pro-260, Ala-306, and Lys-317 on LANA^SIM^ proteins. The donor and receptor of the hydrogen bond are highlighted. Garcimultiflorone H was used as a control.

To further confirm whether both Cambogin and Garcimultiflorone H block the interaction of LANA and SUMO2 through binding to the SIM region, co-immunoprecipitation experiments were performed in HEK293 cells co-expressing myc-tagged LANA and FLAG-tagged SUMO2 in the presence of Cambogin or Garcimultiflorone H. Along with untreated or DMSO as controls, the results showed that Cambogin, but not Garcimultiflorone H, dramatically reduced the association of LANA with SUMO2-modified substrate [SUMO2(n)-sb] with high molecular weight (>100 kDa), but no effect on the association of KAP1 with LANA or SUMO2 (Fig 2A). To confirm this effect, the high purity (>98.4%) of Cambogin was verified by UPLC chromatogram (Fig 2B). In the PEL cells, the interaction of endogenous LANA with SUMO2(n)-sb efficiently inhibited by Cambogin in a dose-dependent manner was also observed (Fig 2C). The similar results from *in vitro* GST-LANA pull-down assays using His-SUMO2 further confirmed that Cambogin directly blocked the interaction of LANA with SUMO2 (Fig 2D, compare lanes 2, 3 with 4, 5). To address the difference between Cambogin and Garcimultiflorone H, molecular docking analysis were carried out and revealed that Cambogin instead of Garcimultiflorone H mainly binds to the Gln-258 and Thr-261 residues within the active center of LANA^SIM^ through hydrogen bonding, in addition to forming hydrophobic interactions with Pro-260, Ala-306 and Lys-317 (Fig 2E and 2F). The result that site mutation of both Gln-258 and Thre-261 (QTA) leads to dramatically loss of Cambogin inhibitory effect on the interaction of LANA with SUMO2 (Fig 2D, compare lane 5 with 6), indicating that the subtle structure of compound is a key for blocking the interaction of LANA with SUMO2.

### Cambogin preferentially targeted KSHV-infected cells at a low concentration

**Fig 3:**
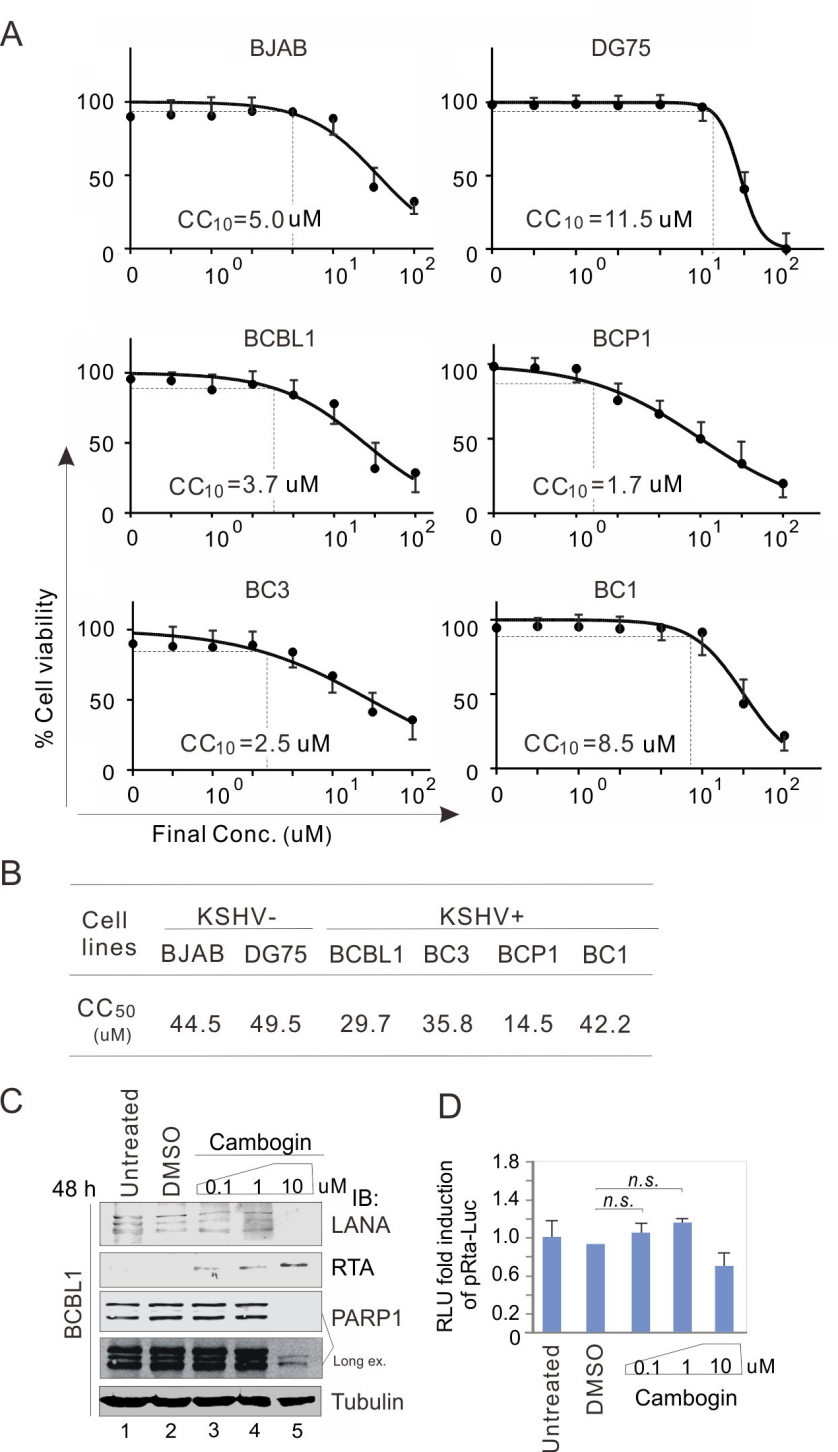
Cambogin presents higher cytotoxicity in PEL cells. (**A**) Cambogin induced relatively higher cytotoxicity in the KSHV-infected cells. The cell viability of KSHV positive and negative B-lymphoma cell lines treatment with different dosage of Cambogin treatment for 72 h. The concentration of Cambogin-induced 10% cell death (CC_10_) was indicated. (**B**) The concentration of Cambogin-induced 50% cell death (CC_50_) was calculated from panel A. (**C**) Cambogin moderately enhances the expression of RTA. BCBL1 cells were treated with DMSO or different concentrations of Cambogin for 48 h, followed by immunoblotting assays as indicated in the figure. Untreated was used as a control. (**D**) Reporter assays of the Rta promoter in the presence of Cambogin. HEK293T cells were transfected with the Rta promoter-driven Luciferase reporter (pRta-Luc). At 24 h post-transfection, cells were treated with DMSO or different concentrations of Cambogin for 24 h, and then harvest for reporter assays. The results were presented by the RLU (relative luciferase unit) fold compared with the untreated group. Data are presented as means±SD of three independent experiments. *n*.*s*., no significant.

Given that Cambogin can have cytotoxicity in both medullobalstoma and breast adenocarcinoma cells at about 50-fold higher concentration by alteration of mitochondrial morphology and dynamics [24,25]. To determine the cytotoxic effects of Cambogin on KSHV-infected cancer cells, we treated BJAB and iSLK cells with or without KSHV infection in the presence of different dosages (0, 0.1, 1, 10, and 100 μM) of Cambogin, along with Garcimultiflorone H, Oblongifolin L, and Heptahydroxy Flavononylflavone as controls for 24 h. The results showed that Cambogin but not Garcimultiflorone H, Oblongifolin L, or Heptahydroxy F exhibited preferential cytotoxicity in KSHV-infected cells in a dose-dependent manner (supplementary S1 Fig). The half cytotoxicity value (CC_50_) of Cambogin in KSHV-infected cells was dramatically lower than in KSHV-uninfected cells (BJAB/K-BJAB is 2.38 *vs* 0.68; iSLK/K-iSLK is 9.42 *vs* 0.6, supplementary S1 Fig). Furthermore, cytotoxicity was tested in different PEL cell lines, including BCBL1, BC3, and BCP1 cells (which are singly infected by KSHV), and BC1 cells (which are dually infected by KSHV and EBV). As there is no appropriate control for PEL cells, we included BJAB and DG75 (KSHV- and EBV-negative B lymphoma cell lines) cells as references. The results from cell viability assays showed that the KSHV-infected B cells presented consistently higher sensitivity (CC_50_ is 14.5~35.8 μM in KSHV+, *vs* 44.5~49.5 μM in KSHV-) than the KSHV-uninfected cells (Fig 3A and 3B). In addition, we also observed that the average dosage of no cytotoxicity (90% cell survival, CC_10_) of Cambogin in the most of KSHV-infected cells is at least higher than 1.5 μM (Fig 3A).

To further confirm the non-cytotoxicity effect of Cambogin on the KSHV-infected cells at a lower concentration (< 1.5 μM), we performed immunoblotting assays to examine the expression levels of PARP1 (the apoptotic marker) in the BCBL1 cells treated with 0.1, 1, and 10 μM dosage of Cambogin. The results showed that the expression of PARP1 was dramatically reduced with 10 μM of Cambogin treatment, but no significantly different at 0.1 or 1 μM concentration (Fig 3C, compare lane 5 with lanes 3 and 4). This indicates that Cambogin did not efficiently induce PEL cell apoptosis at a low concentration (< 1 μM). To answer whether Cambogin impairs the KSHV latent and lytic cycles at the low concentration, we also examined that the expression levels of LANA and RTA (the two key antigens individually encoded by KSHV in latency and lytic replication) in the BCBL1 cell line. The results showed that although Cambogin could greatly inhibit the expression of LANA with induction of RTA expression at 10 μM of treated concentration, it was rarely enhanced by treatment with lower than 1 μM concentration (Fig 3C, compare lane 5 with lanes 3 and 4). In contrast, although a slightly increase of RTA expression in BCBL1 cells upon Cambogin treatment at a lower than 1 μM concentration was observed, the transcriptional activity of Rta promoter was not significantly impaired by Cambogin in HEK293 cells (Fig 3D), indicating that the effect of Cambogin on the expression of RTA in BCBL1 cells at a lower than 1 μM concentration is not directly relied on the regulation at transcriptional level, and could be due to the blockade of LANA-SUMO2 interaction and release of the lytic gene silencing.

### Cambogin inhibited KSHV latent replication and primary infection

**Fig 4:**
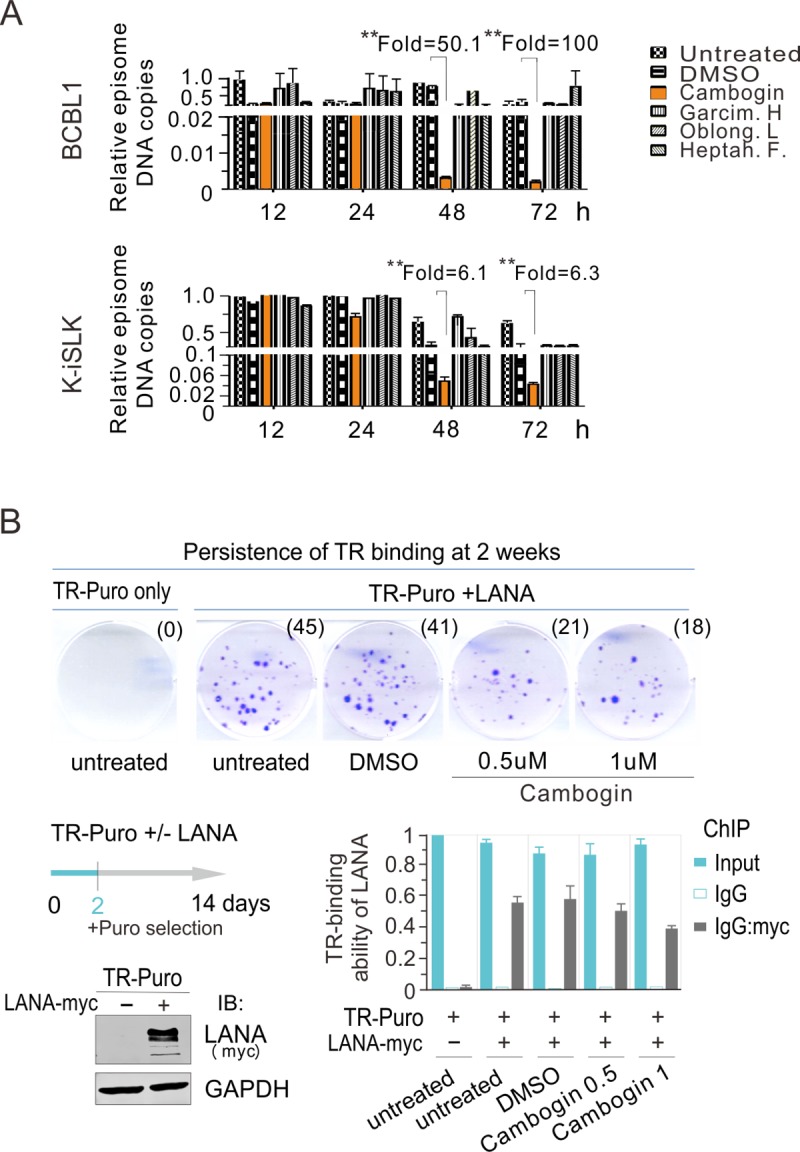
Cambogin blocks maintenance of KSHV episome DNA. (**A**) Equal amounts (0.2 million per ml) of KSHV-infected (BCBL-1, and K-iSLK) cells were individually treated with 100 nM Cambogin, Garcimultiflorone H, Oblongifolin L, Heptahydroxy Flavononylflavone, or DMSO as a control for the indicated time, and then harvested and subjected to quantitative PCR analysis to determine DNA copy number of the KSHV episome. The episome copy number relative to the untreated group was calculated and indicated in the figure. Asterisk indicates a significant difference (*p*<0.01). (**B**) Cambogin did not affect the TR-binding ability of LANA but inhibited the capacity of LANA-mediated TR maintenance. HEK293 cells co-transfected TR-puromycin plasmid with myc-tagged LANA or vector alone were equally divided and treated with different dosage of Cambogin or DMSO. At 48 h post-transfection, cells were subjected to ChIP assays with normal mouse IgG or mouse anti-myc (9E10) antibodies followed by real-time quantitative PCR with TR primer (bottom panels). For colony formation assays of LANA-mediated TR maintenance, three thousands transfected cells at 48 h post-transfection were seed and selected with 2 μg/ml puromycin cells for 2 weeks (Top panels). The average of colonies quantified from duplicate experiments is shown. The expression of exogenous LANA is presented by immunoblotting (IB) assay on the left panel.

To further evaluate the specific effect of Cambogin on the passage of KSHV episome DNA, the KSHV-latently infected cell lines BCBL-1, and K-ISLK were individually subjected to treatment with Cambogin, Garcimultiflorone H, Oblongifolin L, or Heptahydroxy F at 100 nM (a non-cytotoxicity concentration) for 12, 24, 48, and 72 h, followed by quantitative PCR for detection of KSHV DNA. The results showed that Cambogin efficiently decreased KSHV DNA copy number in the KSHV-infected cells after 48 h, when compared to DMSO, Garcimultiflorone H, Oblongifolin L, or Heptahydroxy F (Fig 4A). Since LANA mainly contributes to the maintenance of viral episome during latency, we wanted to know if Cambogin inhibits the persistence of the viral episome during cell passage is through targeting the ability of LANA binding with the terminal repeat (TR). To this end, we performed chromatin immunoprecipitation assays by using myc-tagged LANA co-expressed with the TR-Puromycin plasmid in HEK293 cells with different dosage of Cambogin treatment for 48 h, followed by colony formation assays for 2 weeks. The results showed that although no significantly inhibitory effect between Cambogin and DMSO control treatment in the ability of LANA binding to TR, the Cambogin-treated group presented dramatically inhibition of TR maintenance in a dose-dependent manner (Fig 4B).

**Fig 5:**
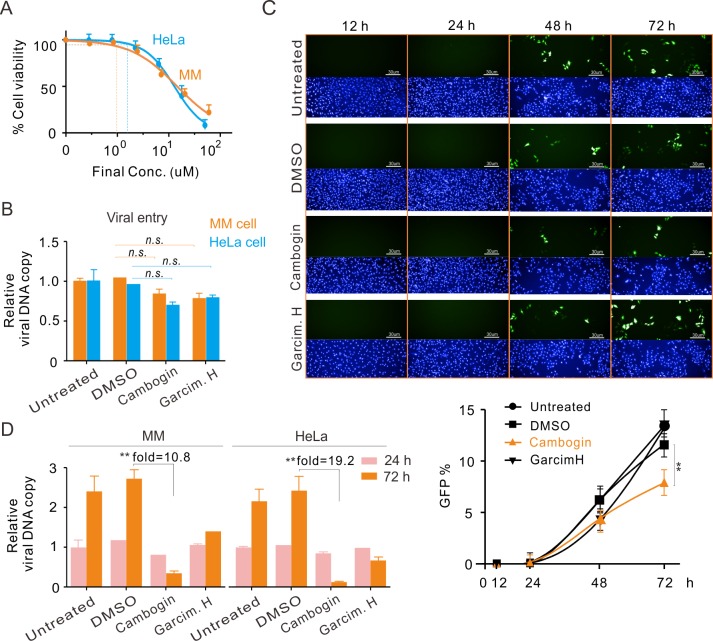
Cambogin inhibits KSHV *de no* infection *in vitro*. (**A**) The cell viability of HeLa and MM cell lines with different dosage of Cambogin treatment for 48 h. The concentration of Cambogin-induced 10% cell death (CC_10_) was indicated. (**B**) Cambogin does not impair viral entry. Equal amount of HeLa or MM cells incubated with GFP-tagged KSHV virions (MOI = 2) in the presence or absence of 100 nM Cambogin and Garcimultiflorone H (DMSO as a control), were subjected to viral entry analysis by quantitative PCR. Asterisk indicates *p*<0.05. *n*.*s*., no significant. (**C**) Cambogin inhibits KSHV *de no* infection. Equal amounts of MM cells with KSHV infection and compounds treatment as described in panel B for 12, 24, 48, and 72 h, were subjected to immunofluorescence analysis of GFP (green) and nuclei staining by DAPI (blue) to detect the infection efficiency of KSHV. The relative percentages were quantified by flow cytometry analysis and shown at the bottom panels. Asterisk indicates *p*<0.05. Similar experiments duplicated in HeLa cells see supplemental **S2 Fig**. (**D**) Cambogin reduces viral episome DNA copy during KSHV primary infection. HeLa and MM cells with KSHV infection and compounds treatment for 24 and 72 h from panel C, were subjected to quantitative PCR analysis for relative KSHV episome DNA copy number. **p < 0.01.

To determine whether Cambogin affects KSHV primary infection, we firstly evaluated the viability of both MM and HeLa cells treated with different dosage of Cambogin. The results showed that the CC_10_ value of both MM and HeLa cells with Cambogin treatment is also higher than 1 μM (Fig 5A). Then, both MM and HeLa cells were individually incubated with GFP-tagged KSHV virions in the presence or absence of 0.1 μM Cambogin for different time points, followed by immunofluorescence and quantitative PCR assays to detect the efficacy of viral entry, infection efficiency and episome copy number, respectively. The results showed that Cambogin did not impair the viral entry, but effectively inhibited the efficiency of KSHV primary infection, and reduced episome DNA copy number, when compared to DMSO or Garcimultiforones H as parallel controls (Fig 5B, 5C and 5D, supplementary S2 Fig). In contrast, no significant effect on HCMV primary infection and virion production were observed (Supplementary S3 Fig), indicating that Cambogin specifically blocked KSHV primary infection.

### Cambogin inhibited the proliferation of diverse PEL and KSHV-transformed cell lines *in vitro*

**Fig 6:**
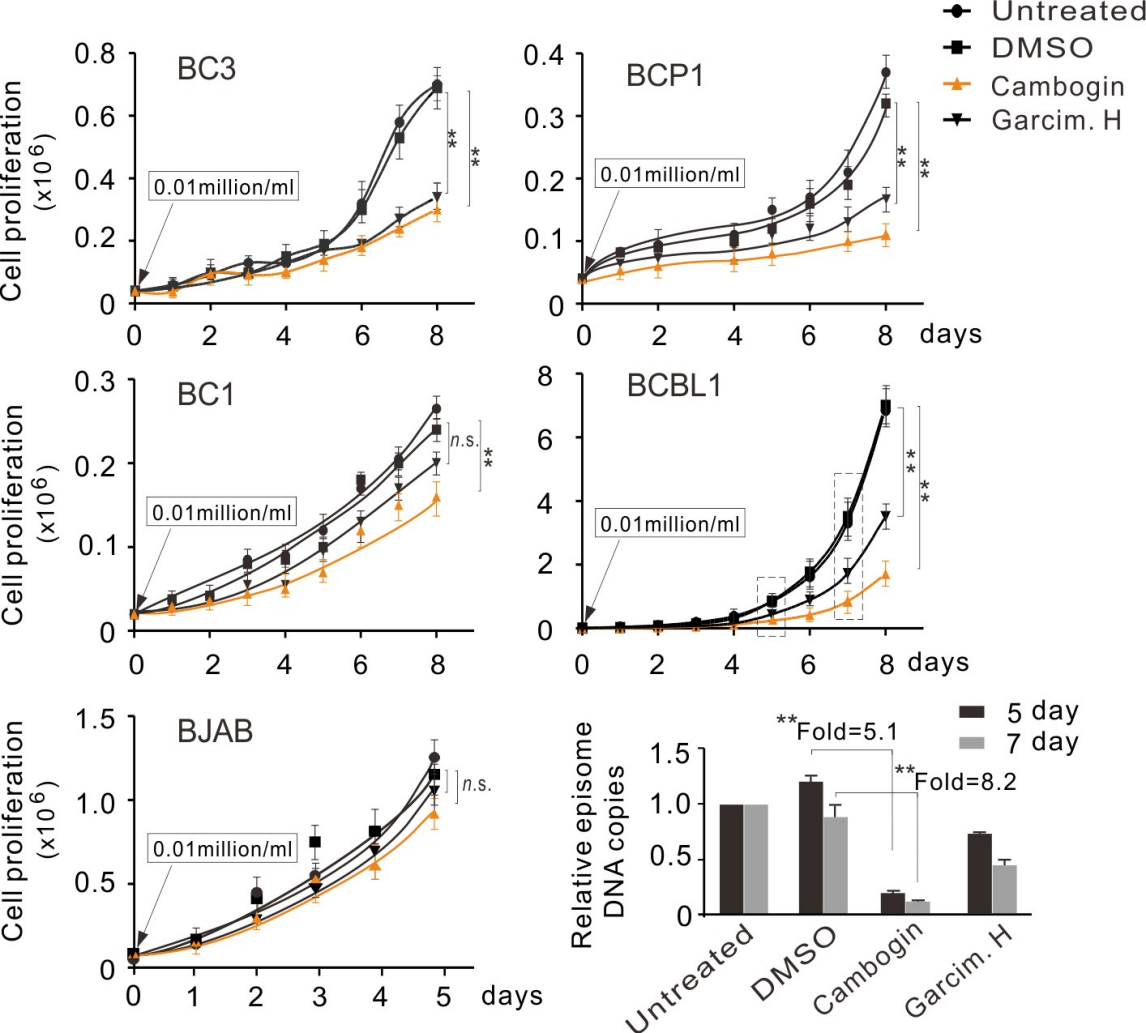
Cambogin dramatically inhibits the proliferation of KSHV-infected cells. Equal amounts of KSHV-positive PEL and KSHV negative BJAB cells were untreated or individually treated with 500 nM Cambogin or Garcimultiflorone H for the indicated time, and then subjected to quantitation of total live cell amount. DMSO was used as a control. The KSHV episome copy number relative to the untreated group at day 5 and 7 in BCBL1 cells was measured and is shown. **p < 0.01; *n*.*s*., no significant.

To further determine the inhibitory effect of Cambogin on KSHV-infected cells, we tested the role of Cambogin on the proliferation of different PEL lines, including BCBL1, BC3, BCP1, and BC1 cells, within 1μM concentration. Consistently with our speculation, at 0.5 μM, Cambogin effectively inhibited the proliferation of all KSHV-positive PEL cells but not KSHV-negative BJAB cell lines, when compared to the DMSO control (Fig 6). Unexpectedly, Garcimultiflorone H also inhibited the proliferation of all PEL cell lines to some extent (Fig 6), albeit it could not efficiently block the interaction of LANA and SUMO2 when compared with Cambogin (Fig 2A). In contrast, the DNA copies of the KSHV genome in BCBL1 cells treated with Cambogin instead of Garcimultiflorone H for 5 and 7 days were significantly lower (5.1-fold and 8.2-fold, respectively) than cells treated with DMSO control in a time-dependent manner (Fig 6, bottom panels). These data further support the notion that Cambogin inhibits the proliferation of KSHV-latently infected cells by disrupting the maintenance of episome DNA.

**Fig 7:**
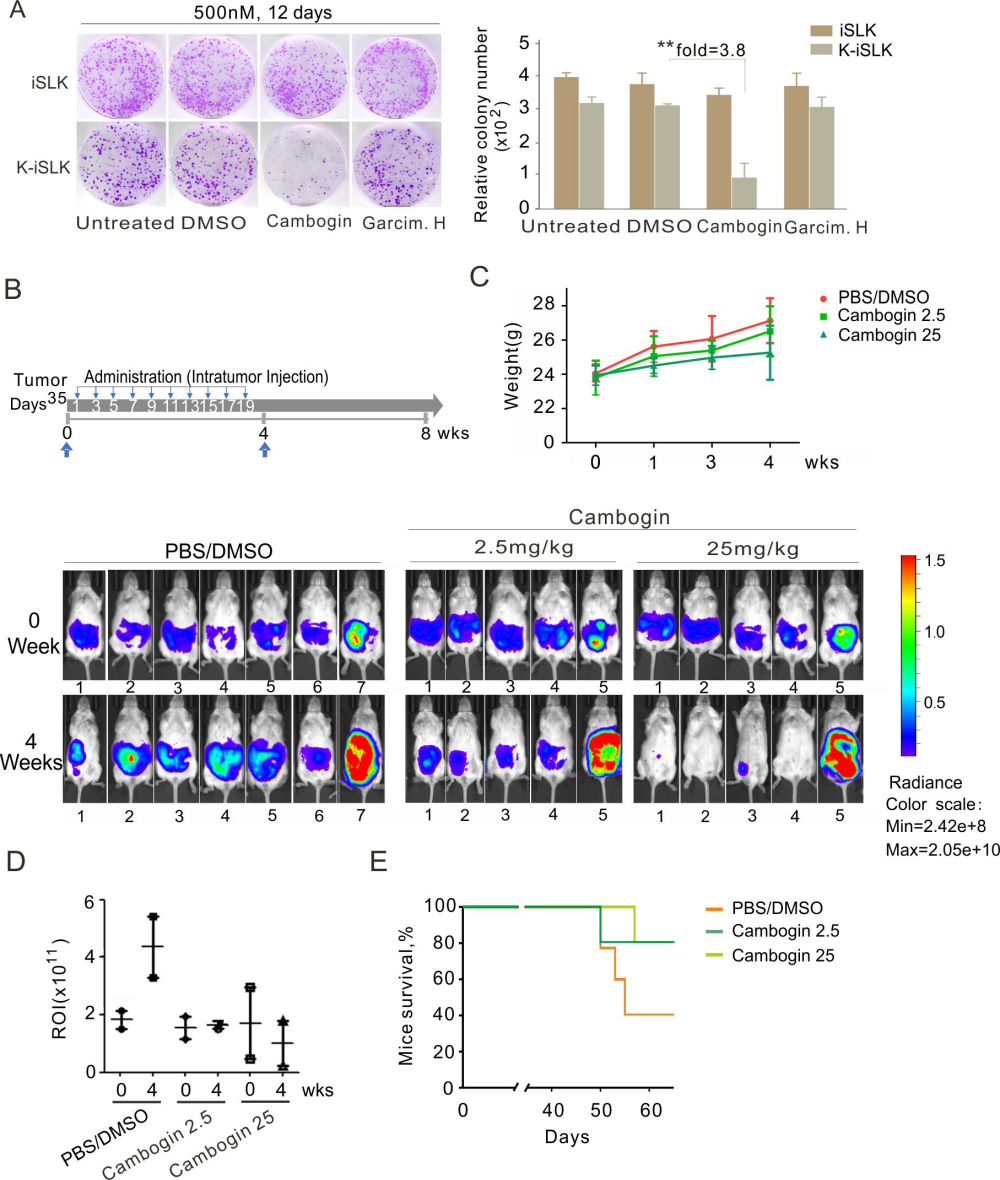
Cambogin significantly inhibits KSHV-latently infected cell growth *in vitro* and i*n vivo*. (**A**) Cambogin selectively reduced the colony formation in KSHV-latently infected cells *in vitro*. Equal amounts of iSLK and KSHV-latently infected iSLK-K cells were individually inoculated and treated with or without Cambogin and Garcimultiflorone H and subjected to colony formation assays as indicated. The cells were fixed 12 days later and stained with crystal violet to determine colony number. A representative well with colony formation is shown. *Right panel*, the relative amount of colony formation was calculated from three independent experiments. Double asterisks indicate *p*<0.01. (**B**) Cambogin inhibited tumor progression in a xenograft mouse model of primary effusion lymphoma. The tumor burden of NOD/SCID mice was analyzed by luminescence assay at week 5 and then following treatment with PBS/DMSO, or Cambogin every other day for 3 weeks. Top panel, schematic of NOD/SCID mice intraperitoneally engrafted with ten million BCBL1-Luc cells for 35 days, and then untreated or treated with same amount of Cambogin every other day for 20 days. (**C**) Weights of NOD/SCID mice intraperitoneally engrafted with 10^7^ BCBL-Luc cells for 5 weeks and then treated with Cambogin, every other day, for 3 weeks, were monitored every week. (**D**) Luminescent signals were quantified and expressed in region-of-interest (ROI) signal intensity [total radiant efficiency(*p*/*s*)/(μW/cm^2^)] in panel B. (**E**) Total percentage of mouse survival in each group is shown in panel B.

To further confirm whether Cambogin effectively inhibits colony formation in KSHV-transformed cells *in vitro*, the KSHV-uninfected and infected cells, iSLK and K-iSLK, were individually treated with or without Cambogin, and then subjected to colony formation assays. Using Garcimultiflorone H and DMSO as parallel controls, the results showed that Cambogin markedly blocked the colony formation of K-iSLK cells but not iSLK cells (Fig 7A). Similar results were observed in the MM and KMM (MM cells with KSHV infection) murine endothelial cells treated with Cambogin, albeit Cambogin also exhibited an inhibitory effect on MM cells to lesser extent (supplementary S4 Fig). Different from the iSLK and K-iSLK cell lines, Garcimultiflorone H inhibited the colony formation of both MM and KMM cells, although it was less effective than Cambogin (Supplementary S4 Fig).

### Cambogin induced regression of PEL tumor growth *in vivo*

To examine the efficacy of Cambogin for the treatment of PEL *in vivo*, a xenograft mouse model was created by engrafting BCBL1-Luc cells into NOD/SCID mice. At day 35 post-engraftment, live bioluminescence imaging was performed to detect tumor signals within each mouse (Fig 7B). The mice exhibiting a similar intensity of tumor signals were randomly separated into groups treated with different-dosages (2.5 and 25 mg/kg) of Cambogin, or PBS/DMSO. After treatment with PBS/DMSO, or Cambogin via intraperitoneally administration, mice began gaining weight as early as 1 week due to PEL development, while those treated with Cambogin maintained a relatively reduced weight (Fig 7C). Consistently, mice treated with Cambogin had a dose-dependent weaker detectable signal at week 4 post-treatment (Fig 7B and 7D), indicating that Cambogin inhibited PEL growth.

Next, we examined whether Cambogin could continuously control regression of PEL tumors after drug withdrawal. At week 8 post-treatment, 4 mice (No. 2, 3, 4, and 7) in the PBS/DMSO group, 1 mouse (No. 5) in the 2.5 mg/kg Cambogin group, and 1 mouse (No. 5) in the 25 mg/kg Cambogin group died of PEL (Fig 7E). Bioluminescence imaging at week 8 post-treatment showed that the remaining 3 mice in the PBS/DMSO group had strong luminescent signals. In contrast, the luminescent signals in all mice treated with Cambogin were dramatically reduced. In particular, those with a higher treatment dose were reduced to almost undetectable levels, indicating that Cambogin effectively induced regression of most of the tumors. These results demonstrated that Cambogin may be an effective drug for inhibiting PEL progression and inducing the regression of PEL.

## Discussion

PEL, as a KSHV-associated malignant B lymphoma, is highly resistant to conventional chemotherapies [26], and currently there are no efficient and specific treatments. To determine the specific cytotoxicity of compounds against PEL cells, many studies have focused on the screening of small molecule compounds by targeting cellular signaling pathways within KSHV latency or inducing reactivation of the lytic cycle [8–15]. However, the effectiveness of these compounds requires further evaluation in addition to consideration of their side effects. Therefore, identification of more effective therapeutic candidates against KSHV is urgently needed. Based on previous data showing that LANA^SIM^ is essential for the establishment and maintenance of KSHV latency, a live-cell based reporter system was developed to screen inhibitors selectively targeting the interaction between LANA^SIM^ and SUMO2. Through this study, a small molecule compound named Cambogin was identified. Cambogin exhibited an efficient inhibitory effect on cell growth in PEL *in vitro* and *in vivo* through specifically reducing DNA copy number of the viral episome in the establishment and maintenance of KSHV latent infection. Therefore, Cambogin may be a promising candidate for the further development of a therapeutic strategy for PEL. To our knowledge, this is the first report to identify a small molecule inhibitor that disrupts KSHV latent infection by directly targeting a viral nuclear antigen important to the maintenance of the viral episome DNA. However, although we did observe that Cambogin efficiently reduces the persistence of LANA-mediated TR and viral episome, whether the recruitment of the Origin Recognition Complex (ORC) and the Mini Chromosome Maintenance (MCM) complex to the viral TR region is affected by Cambogin treatment need to be further investigation.

Increasingly, natural products have indispensably contributed to the discovery and development of effective drugs for the treatment of human malignancies. Emerging evidence has shown that PEL is particularly susceptible to treatment with some natural products. For example, both capsaicin and apigenin are major bioflavonoids present in several vegetables that are consumed daily and have anti-inflammatory and anticancer properties via the interruption of multiple cancer-related pathways including PI3K/AKT and STAT3. In PEL cells, capsaicin not only decreased the expression of the latent and lytic KSHV protein, but also up-regulated the surface expression of HLA-DR for detection by the immune system [27]. In contrast, apigenin was shown to activate p53 and reduce ROS production [28]. Epigallocatechin-3-gallate (EGCG), as a major constituent of green tea, has also been shown to suppress KSHV replication and induce apoptosis and autophagy in PEL cells [29]. Triptolide, a diterpenoid triepoxide purified from the roots of the Chinese herb *Tripterygium wilfordii*, was shown to decrease LANA expression and the viral titer of PEL cells [30]. In addition, Fumagillin, extracted from *Aspergillus fumigatus* and a potent natural angiogenesis inhibitor, also induced KSHV reactivation and inhibited PEL cell growth [31,32]. Thus, a novel inhibitor specifically targeting KSHV-associated tumor cells would provide an effective strategy for the treatment of PEL. To identify the natural product inhibitors specifically blocking the interaction of LANA^SIM^ and SUMO2, we also screened the 73 Chinese herbal compound voucher specimens and further narrowed down those small molecules with well-known structures. Interestingly, we found that Cambogin, a bioactive natural product isolated from the *Garcinia genus* (a traditional herbal medicine used for cancer treatment), efficiently inhibited the association of SUMO2 with LANA^SIM^ at an nM-level concentration, and specifically suppressed the growth of KSHV-infected cells, but not the uninfected cells. Although some previous studies have shown that Combogin induced caspases-independent apoptosis in breast cancer cells [24,33], and is preferentially cytotoxic to cells expressing PDGFR [25], it exerts non-cytotoxicity to B-lymphoma cells, including KSHV-infected PEL cells, at a low concentration of less than 1 μM. More importantly, Cambogin markedly inhibits the *in vitro* proliferation of different types of KSHV-infected cells (PEL, KMM, K-iSLK), instead of the KSHV-uninfected cells (BJAB, MM, iSLK). These results show the specific anti-tumor activity and favorable therapeutic index of Cambogin in the KSHV-infected cancers. The xenograft mouse model experiment results further demonstrate that Cambogin is a preferential drug for the inhibition of PEL tumor regression. However, it was also observed that Cambogin did not effectively induce regression of PEL with a large tumor size, although the overall survival of tumor xenograft mice was extended. This result indicates that Cambogin exerts greater inhibitory effect in the early stage than in the late stage of PEL development. In addition, it is worthy to mention that Cambogin is administered intraperitoneally for inhibiting the PEL growth *in vivo* in this study, how well Cambogin does when administered orally remains unclearly and needs to be further investigation.

Combination cytotoxic chemotherapy is the current standard of care for PEL [5,6], however, lack of efficacy, off-target effects, and chemotherapeutic resistance continue to limit the utility of this approach. A combination of highly active anti-viral therapies is still being explored [12]. In the current study, in addition to Cambogin, Garcimultiflorone H (which has a similar molecular structure) exhibited an inhibitory effect on the association of SUMO2 with LANA^SIM^ in the LgBIT-SUMO2/LANA-SmBIT-screening system. However, Garcimultiflorone H did not effectively inhibit the establishment and maintenance of KSHV latent episome DNA. Although the inhibitory effect of Garcimultiflorone H on the proliferation of PEL cells was lower than Cambogin, it was still much higher than the DMSO control. This indicates that Garcimultiflorone H may be inefficiently in blocking the interaction of SUMO2 with LANA^SIM^ due to the subtle difference in molecular structure compared to Cambogin. Interestingly, Garcimultiflorone H also exhibited a similar effect as Cambogin on the inhibition of EBV-infected B cell growth. Given the fact that most AIDS-associated PEL malignancy is associated with KSHV and EBV infection, the combination of Garcimultiflorone H with Cambogin could provide another effective strategy for inhibiting the growth of PEL. However, this requires further investigation.

Most KSHV-associated cancer cells harbor a latent infection, and the few cells that undergo lytic replication may contribute to pathogenesis and tumorigenesis. Additionally, the cytotoxic drugs used to treat cancers harboring viral infection often contribute to activation of the viral lytic cycle as a side effect. Numerous studies have shown that the targeting of pathways involved in differentiation and survival is a promising strategy for treating PEL. For example, Nutlin-3 induced apoptosis in PEL cells through disruption of p53-MDM2 interaction [34]. Triptolide inhibits cell proliferation and PEL progression by suppressing STAT3 activity, IL-6 secretion, and LANA expression [30]. Our study revealed that Cambogin mainly disrupts the maintenance of KSHV latent episome DNA and establishment during primary infection, with less reactivation of the lytic cycle. This is a potential explanation for the effective regression of PEL following Cambogin treatment *in vivo*. Taken together, our studies have identified Cambogin as a promising candidate for the treatment of PEL patients.

## Materials and methods

### DNA constructions and reagents

Plasmids expressing recombinant proteins of LANA1-329-WT, LANA1-329-ΔSIM or SUMO2(ΔGG) with LgBiT or SmBiT were generated by PCR amplicon inserted into the vectors of pBiT1.1-C[TK/LgBiT], pBiT2.1-C[TK/SmBiT], pBiT1.1-N[TK/LgBiT] and pBiT2.1-N[TK/SmBiT] with restriction enzymes *Bgl*II and *Eco*RI, respectively. Plasmids LANA-myc, LANA_1-329_-WT or ΔSIM, FLAG-SUMO2, GST-LANA_1-329_(N) and His-SUMO2 were described previously [23,35]. GST-LANA_1-329_(N) with Q258A and T261A double mutation was constructed by PCR-direct site mutation technique. Antibodies to PARP1 (F2, Santa cruz), KAP1 (20C1, Abcam), FLAG (M2, Sigma), His (10E2, Abmart), SUMO2 (EPR4602, Abcam), GAPDH (60004-1-Ig, Proteintech) and Tubulin (66031–1, Proteintech) were used according to the manufacturers specifications. Mouse monoclonal antibodies against LANA1, RTA or myc (9E10) were stored in the lab. Protease inhibitors phenylmethylsulfonyl-fluoride (PMSF), Leupeptin, Aprotinin, and Pepstatin A, and SENP inhibitor N-ethyl-maleimide (NEM) were purchased from Amresco.

Seventy-three Chinese herbal compounds voucher specimens were deposited at the Engineering Research Center of Shanghai Colleges for TCM New Drug Discovery, Shanghai University of Traditional Chinese Medicine. Cambogin was isolated from the twigs of Garcino esculentat Y. H. Li, which collected in Nujiang, Yunnan Province, China, in August 2010. Cambogin’s structure was determined using ^1^H-NMR and ^13^C-NMR spectral analysis, and the purity of this compound was more than 98% based on HPLC analysis (Fig 2B). Ultra-high performance liquid chromatography (UPLC) was performed using a Waters Acquity UPLC (Waters, Milford, MA, USA), equipped with a Quaternion solvent delivery system, an autosampler and a photodiode array detection system. Chromatography was used a Waters ACQUITY UPLC HSS T3 (1.7 μm, 2.1*100 mm). The mobile phase consisted of acetonitrile (A) and 0.1% formic acid (B). And the UPLC eluting conditions were as follows 0–15 min, 60%-90% (A); 15–18 min; 90%-100% (A); 18–20 min, 100–60% (A). Detection wavelength: 232nm, 278nm, 315nmm; flow rate: 0.4 mL/min; inject volume: 2 μL. Cambogin was dissolved in absolute dimethyl sulfoxide (DMSO) at a concentration of 10 mM for in vitro experiments (100 mM for *in vivo* experiments), then diluted in tissue culture medium and filtered before use. The 0.5% DMSO in cell experiments was used as a control.

### Cell lines

KSHV and EBV-double negative BJAB and DG75 (American Type Culture Collection, ATCC), KSHV-positive BC3, BCBL1, and BCP1, and KSHV and EBV-double positive BC1 cells were cultured in RPMI 1640 supplement with 10% fetal bovine serum (FBS, Hyclone). iSLK (1μg/ml puromycin, 250μg/ml G418) and K-iSLK (1mg/ml hygromycin, 250μg/ml G418 and 1μg/ml puromycin, a gift from Shou-Jiang Gao at University of Pittsburgh) cells were maintained in Dulbecco’s modified Eagle’s medium (DMEM) containing 10% FBS. Rat primary embryonic mesenchymal stem cells (MM) and KSHV-transformed MM cells (KMM), a gift from Shou-Jiang Gao at University of Pittsburgh, were maintained in DMEM containing 10% FBS. Human embryonic lung fibroblasts MRC5, Hela and HEK293 cells were routinely cultured in DMEM containing 10% FBS. All mediums were supplement with 100 U/ml penicillin and 100 μg/ml streptomycin. All cell lines were incubated at 37°C in a humidified environmental incubator with 5% CO_2_.

### Luciferase assay

HEK293 cells were plated at 40,000 cells per well in 24-well plate for 24 h with cell confluence reaching 60–70%. Cells were transfected Nano BiT vectors (Promega, Cat.# 2014) with FuGENE HD Transfection Reagent (Promega, Cat.# E2311) according to the manufacturer's recommendations. HEK293 were incubated at 37°C in a 5% CO_2_ incubator for 6 h, and then aspirated medium and replaced with new medium. Nano-GloR LCS Dilution Buffer need to be equilibrated at ambient temperature and Nano-GloR Live Cell Substrate were diluent by LCS (1:20). Aspirate medium and replace with 100μl of cell culture medium to harvest cells, supernatants were then transferred to a new 96-well white plate, and added 25μl of Nano-GloR Live Cell Reagent to each well, and gently mix the plate by hand and then wait 15–20 min to ensure thermal equilibration of the entire plate at 37°C, and measure luminescence by microplate reader (Tecan). A pair of strongly interacting proteins (LgBIT-PRKAR2A/SmBIT-PRKACA) is used as a positive control, and the HaloTag-SmBiT and LgBIT-SUMO2 constitutes as a negative control.

### Small compounds screening of Chinese herbal extracts

HEK293 cells were seeded in 24-well plated at 40,000 cells per well for 24 h and then transfected LANA WT-SmBiT and LgBiT-SUMO2 at ratio 1:1 with FuGENE HD transfection reagent. At 6 h post-transfection, cells were treated with small compounds at 100nM final concentrations in DMSO for 48 h, and then measured by luciferase assay. DMSO was used as a parallel control. The results were analyzed with graphpad prism 5.0 software. The luminescence of live cells treated with DMSO was set as control. Compounds that showed at least 50% inhibition to the interaction of LANA and SUMO2 were chosen.

### Immunoprecipitation and immunoblotting

HEK293 cells were transfected with 1 mg/ml polyethyleneimine (PEI) at a ratio of 5μg LANA-myc and 5μg FLAG-SUMO2: 30μl PEI. At 6 h post-transfection, cells were treated with small molecules at 100 nM final concentrations in DMSO for 48 h. DMSO and PBS were used as parallel controls. Cell were harvested, washed once with ice-cold PBS, and lysed in 600 μl ice-cold RIPA buffer [150mM NaCl, 50mM Tris (pH7.6), 1% Nonidet P-40, 2 mM EDTA, 1mM PMSF, 1g/ml aprotinin, 1g/ml leupeptin, 1 g/ml pepstatin, 2.5mM NEM] for 30 min with constant vortex at 4°C. Cell debris was removed by centrifugation at 14,500 rpm at 4°C for 10min. The supernatants were then transferred to a new eppendorf tube. Five percent of the supernatant was used as input. The rest lysates were then precleared with normal mouse IgG (Invitrogen) and protein A/G Sepharose beads by end-over-end rotation at 4°C for 1 h. After preclear, beads were spun out. Supernatant was then incubated with primary antibody at 4°C with rotation overnight. Protein of interest complexes were captured the next day with 30μl protein A/G Sepharose beads with rotation on 4°C for another 4 h. Beads were spun out, washed with ice-cold RIPA buffer for four times and re-suspended with 30μl RIPA buffer. For immunoblotting assays, the input lysates and immunoprecipitated (IP) complexes were boiled in 6xSDS loading buffer, proteins were fractionated by SDS-PAGE, and transferred to a 0.45-mm nitrocellulose membrane. The protein of interest in the membrane was probed with primary antibodies at 4°C with shake for overnight, followed by incubation with appropriate secondary antibodies for another 1 h at room temperature. The member was scanned with an Odyssey Infrared scanner (Li-Cor Biosciences). Densitometric analysis was performed with the Odyssey scanning software. The membrane was stripped using stripping buffer (200 mM Glycine, 1% SDS, pH 2.5) for re-immunoblotting.

### GST pull-down assay *in vitro*

Overnight starter cultures (50 ml) of BL21 (DE3) transformed with plasmid expressing His-SUMO2, GST or GST-LANA_1-329_ protein were used to individually inoculated 500 ml of Luria Broth (LB) culture medium with specific antibiotic and grown at 30°C to a density of appropriately 0.6 optical density at 600nm. After 1 mM isopropylthiogalactopyranoside (IPTG) induction at 30°C for 4 h, the bacteria were collected and sonicated in lysis buffer (20 mM Tris-HCl pH 8.0, 100 mM NaCl, 0.5% NP40, 1 mM EDTA, 1 M DTT, 5% Sarkosyl and the protease inhibitor cocktail). Recombinant proteins His-SUMO2 and GST-LANA_1-329_ were purified by Glutathione Sepharose and Ni^2+^ beads, respectively. For pull-down assay, purified His-SUMO2 proteins were individually incubated with GST or GST- LANA_1-329_ proteins loaded on beads for 3 h at 4°C in NETN binding buffer (50 mM Tris-HCl pH 7.5, 100 mM NaCl, 10 μm ZnCl_2_, 10% glycerol, freshly supplemented with 0.1 mM Dithiothreitol and protease inhibitors). After washing, unbound and bound proteins were individually eluted with SDS sample buffer and analyzed by gel electrophoresis followed by immunoblotting assays.

### Interaction mode analysis of LANA^SIM^ and Cambogin

The three-dimensional structure of LANA^SIM^ domain (233–240 aa) is used as a molecular docking protein [23], which is hydrogenated by SYBYL software, charged by Biopolymer/prepare structure/Load charge, and optimized by Computer/Minimize Staged minimization. Three-dimensional conformation, optimization and format conversion of docking compounds cambogin is prepared. The coordinates of the docking center are X = -9.627, Y = -15.684, Z = 50.539, and the radius of the docking is 20Å. The template for docking is chemscore kinase, and the scoring function for docking is ChemPLP. Results of the conformation of 10 small molecules were retained.

### Cell viability assay

Cells were seeded at a density of 0.2 million per ml, and treated with different small compound at a series of concentration (1, 10, 100μM) for 48 h. B-lymphoma cells viability was real-time measured by Live cell counter (Beckman) with trypan blue staining. For iSLK and K-iSLK cells, cells viability was detected by Methyl-thiazolyltetrazolium (MTT) solution (5 mg/ml, Sigma) as described previously [36].

### Quantitation of viral episome DNA

Total DNA was extracted by lysing buffer (10 mM Tris-HCl pH 8.0, 150 mM NaCl, 10 mM EDTA, 1% SDS) followed by proteinase K digestion. The numbers of KSHV episomal DNA copies were calculated by quantitative PCR targeting ORF72 (sense: 5’-GTTCCACTGCCGCCTGTA-3’, anti-sense: 5’-TATTTGGGACCTTTCAACAA TCTCTT-3’, 609bps). The pET-28a plasmid carrying ORF72 DNA was used as a standard control. Actin (sense: 5’-CTCCATCCTGGCCTCGCTGT-3’; anti-sense: 5’-GCTGTCACCTTCA CCGTTCC-3’, 268 bps) was used as an internal control. Relative gene expression levels were calculated by using the threshold cycle (2^_ΔΔ*CT*^) formula.

### Chromatin immunoprecipitation

The chromatin immunoprecipitation (ChIP) experiments were performed as previously described [23]. Briefly, cells (3×10^8^) were cross-linked with formaldehyde in growth medium at 37°C for 10 min, and then at 4°C for 50 min. Formaldehyde was quenched by adding glycine. Fixed cells were washed with PBS, and sonicated to an average fragment size of 300–500 bp. Solubilized chromatin extracts were clarified by centrifugation, diluted and pre-incubated with protein A-Agarose (Invitrogen Life Technologies) with normal mouse sera. Aliquots (600 μl) were incubated with 20 μg of anti-myc (9E10) antibody for overnight at 4°C. Immune complexes were separated into bound and unbound complexes with protein A-agarose and cross-links were reversed by treatment at 65°C overnight. After treatment with RNase A and proteinase K, samples were extracted once with phenol/chloroform, and the DNA was precipitated. Precipitated DNA was pelleted, washed once with 70% ethanol, dried, and resuspended in distill water. The DNA was analyzed by PCR using TR primer. Relative enrichment of DNA binding was shown as a percentage of input.

### KSHV virion purification and primary infection

The K-iSLK cells were subjected to induction for KSHV reactivation by Doxycyclin (1ug/ml) and sodium butyrate (1mM). After induction, the supernatant of culture medium was collected and filtered through 0.45 mm filter, and viral particles were spun down at 25,000 rpm for 2 h at 4°C. The concentrated virus was collected and used for primary infection. Hela cells (1.5x10^5 per well in 24-well plate) infected by centrifugation at 1,500 rpm at 25°C for 1 h, and then returned to 37°C for 1 h with 2% FBS culture medium. After washing with PBS for three times, compounds was added at a concentration 100nM. Infection efficiency was measured by flow cytometer and immunofluorescence assay.

### Viral entry assay

MM and Hela cells (1x10^4^ per well in 96-well plate) that incubated with GFP-tagged KSHV virion particles (MOI = 5) generated from K-iSLK cells for 2 h on ice, were washed twice with PBS to remove unbound virus, and then returned to 37°C for 1 h with 6% FBS culture medium with 100nM compounds. The cells were digested with 0.05% trypsin-EDTA at 37°C for 5 min to remove non-internalized virus, and washed twice with PBS and lysed immediately for KSHV DNA extraction and quantitative PCR analysis by using ORF73 as target.

### HCMV primary infection

Bacterial artificial chromosome (BAC) pAD-GFP carried the green fluorescent protein (GFP)-tagged genome of the HCMV AD169 strain was used to produce wild-type virus ADwt as described previously[37]. Human embryonic lung fibroblast MRC5 cells were seeded at a density of 8,000 cells per well in 96-well plates and allowed to attach overnight. Cells were infected with wild-type HCMV at a multiplicity of infection (MOI) of 0.3 or 3 in DMEM containing 10% FBS for 2 h, (at this point, collected cell supernatant and virus titer was used as input), and then replace the normal medium (500μl) and add DMSO and Cambogin (0.5 and 1μM). MRC5 cells were examined under a phase-contrast microscope at 4 day or 8 day post-infection (dpi) for their infection efficiency. Dying cells were detached from the culture plate and fragmented, while surviving cells remained attached and maintained a fibroblast-like morphology. At the indicated time points, collected supernatant was used to analysis for HCMV infection efficiency by TCID50 in human fibroblast cells.

### Cell proliferation and colony formation assay

Equal amount of cells were seed and treated with 100nM Cambogin or Garcim.H. Cells growth rate was real-time measured by live cell counter (Beckman) with trypan blue staining. Experiments were performed in triplicate. For colony formation, equal amount of cells were dispersed to 10-cm plate and then treated with 100nM Cambogin or Garcim. H (Every three days change medium and add new compounds). After 2 weeks, cell culture supernatants were discarded and fixed with 4% (v/v) formaldehyde, and then stained with 0.1% crystal violet. Colony formation in each dish was scanned by Li-Cor Odyseesy and counted by Image J. Experiments were performed in triplicate.

### Animal experiments

For the tumor formation experiment, 30 female NOD/SCID mice (Beijing Vital River Laboratory Animal Technology Co., China) at 5 weeks old were each intraperitoneally injected with 10^7^ BCBL1 cells expressing luciferase (BCBL1-Luc). At week 5 post-inoculation, mice were intraperitoneally injected with D-luciferin at 150 mg/kg body weight and imaged with an IVIS Spectrum in vivo imaging system (PerkinElmer). The region-of-interest (ROI) signals based on the (p/s)/(microwatts/square centimeter) formula were analyzed with Living Image software (PerkinElmer). In our preliminary experiments, we found that the toxicity of Cambogin was negligible at 25 mg/kg *in vivo*. For the tumor regression experiment, 17 NOD/SCID mice with similar size were randomly split into 4 groups, and individually treated with PBS, DMSO, 2.5 mg/kg and 25 mg/kg every other day for 3 weeks, and all groups were weighed once a week. At week 9 post -inoculation (namely, week 4 post-treatment), live imaging was performed.

### Ethics statement

All animal studies were conducted in accordance with China guide for the Care and Use of Laboratory Animals. All experiments were approved and overseen by the institutional animal care and use committee at Fudan University under Protocol ID 196086.

### Statistical analysis

The statistical analysis was performed by using SPSS software. The experimental and control groups were assessed by student’s *t*-test for single comparisons. *P* values less than 0.05 were considered to indicate statistically significant differences.

## Supporting information

S1 Table Information of Chinese herbal compounds. (PDF)

S1 Fig Cambogin induced relatively higher cytotoxicity in the KSHV-infected cells. The cell viability of KSHV-infected and uninfected BJAB and iSLK cells treated with different dosage of compounds for 24 h as indicated in figure. The concentration of Cambogin-induced 50% cell death (CC_50_) was calculated and shown.
(TIF)

S2 Fig Cambogin reduces viral episomal DNA copy in HeLa cells with KSHV *de no* infection. Equal amounts of Hela cells incubated with GFP-tagged KSHV virions (MOI = 2) were untreated or treated with 100 nM Cambogin and Garcimultiflorone H (DMSO as a control) for 12, 24, 48, and 72 h, followed by immunofluorescence analysis to detect the infection efficiency of KSHV.
(TIF)

S3 Fig Cambogin did not impair the primary infection of HCMV. Equal amounts of MRC5 cells incubated with GFP-tagged HCMV (MOI = 0.3 or 3) were subjected to similar treatment and analysis at day 4 or 8 post-infection as described in Fig 5D. The virion particles of TCID50 from supernatants of cells with MOI = 0.3 are shown at the bottom panel. *p > 0.05.
(TIF)

S4 Fig Cambogin selectively reduced the colony formation in KSHV-latently infected MM cells *in vitro*. Equal amounts of MM and KSHV-latently infected KMM cells were individually inoculated and treated with or without Cambogin and Garcimultiflorone H and subjected to colony formation assays as indicated. The cells were fixed 10 days later and stained with crystal violet to determine colony number. A representative well with colony formation is shown. *Bottom panels*, the relative amount of colony formation was calculated from three independent experiments. **p < 0.01.
(TIF)
