# Supplementary material for: Identification of viral SIM-SUMO2-interaction inhibitors for treating primary effusion lymphoma
Source: PLoS Pathog. 2019 Dec 12;15(12):e1008174. doi: 10.1371/journal.ppat.1008174 (PMC6932820; doi:10.1371/journal.ppat.1008174)
Supplement: S1 Table — (PDF) [file ppat.1008174.s001.pdf]

Table S1. Information of chinese herbal compounds

| No. | Name | Extract solution | Source (structure type) | M. W. |
|---|---|---|---|---|
| 1 | Ge23(GF): guttiferone F | Column chromatography | *Garcinia esculenta (PPAP)* | 602 |
| 2 | Ge6：cambogin | Column chromatography | *Garcinia esculenta (PPAP)* | 602 |
| 3 | Gm18-4：garcimultiflorone H | Column chromatography | *Garcinia multiflora (PPAP)* | 534 |
| 4 | Gof8：oblongifolin L | Column chromatography | *Garcinia oblongifolia (PPAP)* | 502 |
| 5 | 1,3,6,7-tetrahydroxyxanthone | Column chromatography | *Garcinia esculenta (Xanthones)* | 260 |
| 6 | Ge23: garcinol | Column chromatography | *Garcinia esculenta (PPAPs)* | 540 |
| 7 | OC：oblongifolin C | Column chromatography | *Garcinia yunnanensis (PPAP)* | 670 |
| 8 | GK：guttiferone K | Column chromatography | *Garcinia yunnanensis (PPAP)* | 602 |
| 9 | GA：gambogic acid | Column chromatography | *Garcinia hanburyi (caged xanthones)* | 628 |
| 10 | A-4-a：Oliganthin I | Column chromatography | *Garcinia oligantha (Xanthones)* | 478 |
| 11 | 09-4-2：volkensiflavone | Column chromatography | *Garcinia yunnanensis (biflavonoids)* | 464 |
| 12 | E-7：heptahydroxy[I-3,II-8]flavononylflavone | Column chromatography | *Garcinia(Xanthones)* | 556 |
| 13 | N7：nujiangexanthone A | Column chromatography | *Garcinia nujiangensis (Xanthones)* | 494 |
| 14 | Yb(1)：neobractatin | Column chromatography | *Garcinia bracteata (caged xanthones)* | 464 |
| 15 | Ypa：isobractatin | Column chromatography | *Garcinia bracteata (caged xanthones)* | 464 |
| 16 | A-4-b (A-3-2-2)：Gaudichaudione H | Column chromatography | *Garcinia oligantha (caged xanthones)* | 602 |
| 17 | Ge3：Griffipavixanthone | Column chromatography | *Garcinia esculenta (bixanthones)* | 652 |

| | | | |
|---|---|---|---|
| 18 | Spica prunellae extract | Ethanol | *Spica prunellae* |
| 19 | CXF-5 | Petroleum ether | *Anemarrhena asphodeloides Bge .* |
| 20 | CXF-6 | Ethyl acetate | |
| 21 | CXF-7 | Ethanol | |
| 22 | CXF-8 | H2O | |
| 23 | CXF-177 | Petroleum ether | *Woodwardia japonica(l.f.) Smith* |
| 24 | CXF-178 | Ethyl acetate | |
| 25 | CXF-179 | Ethanol | |
| 26 | CXF-180 | H2O | |
| 27 | CXF-181 | Petroleum ether | *Sophora flavescens Ait.* |
| 28 | CXF-182 | Ethyl acetate | |
| 29 | CXF-183 | Ethanol | |
| 30 | CXF-184 | H2O | |
| 31 | CXF-185 | Petroleum ether | *Sanguisorba officinalis L.* |
| 32 | CXF-186 | Ethyl acetate | |
| 33 | CXF-187 | Ethanol | |
| 34 | CXF-188 | H2O | |
| 35 | CXF-189 | Petroleum ether | *Forsythia suspensa(Thunb.) Vahl* |
| 36 | CXF-190 | Ethyl acetate | |
| 37 | CXF-191 | Ethanol | |
| 38 | CXF-192 | H2O | |

| | | | |
|---|---|---|---|
| 39 | CXF-201 | Petroleum ether | *Baphicacanthus cusia (Nees)Bremek.* |
| 40 | CXF-203 | Ethanol | |
| 41 | CXF-204 | H2O | |
| 42 | CXF-209 | Petroleum ether | *Isatis indigotica Fort.* |
| 43 | CXF-210 | Ethyl acetate | |
| 44 | CXF-211 | Ethanol | |
| 45 | CXF-212 | H2O | |
| 46 | CXF-213 | Petroleum ether | *Lonicera japonica Thunb.* |
| 47 | CXF-214 | Ethyl acetate | |
| 48 | CXF-215 | Ethanol | |
| 49 | CXF-216 | H2O | |
| 50 | CXF-217 | Petroleum ether | *Platycladus orientalis(L.)Franco* |
| 51 | CXF-218 | Ethyl acetate | |
| 52 | CXF-219 | ethanol | |
| 53 | CXF-220 | H2O | |
| 54 | CXF-221 | Petroleum ether | *Fraxinus stylosa Lingelsh.* |
| 55 | CXF-222 | Ethyl acetate | |
| 56 | CXF-223 | Ethanol | |
| 57 | CXF-224 | H2O | |
| 58 | CXF-225 | Petroleum ether | *Eugeia caryophyllata Thunb.* |
| 59 | CXF-226 | Ethyl acetate | |
| 60 | CXF-227 | Ethanol | |
| 61 | CXF-228 | H2O | |

| | | | |
|---|---|---|---|
| 62 | CXF-229 | Petroleum ether | *Terminalia chebula Retz.* |
| 63 | CXF-230 | Ethyl acetate | |
| 64 | CXF-231 | Ethanol | |
| 65 | CXF-232 | H2O | |
| 66 | CXF-549 | Petroleum ether | *Sophora japonica L.* |
| 67 | CXF-550 | Ethyl acetate | |
| 68 | CXF-551 | Ethanol | |
| 69 | CXF-552 | H2O | |
| 70 | CXF-565 | Petroleum ether | *Pulsatilla chinensis ( Bge. ) Regel* |
| 71 | CXF-566 | Ethyl acetate | |
| 72 | CXF-567 | Ethanol | |
| 73 | CXF-568 | H2O | |
