# Supplementary figures and images for: Identification of viral SIM-SUMO2-interaction inhibitors for treating primary effusion lymphoma
Source: PLoS Pathog. 2019 Dec 12;15(12):e1008174. doi: 10.1371/journal.ppat.1008174 (PMC6932820; doi:10.1371/journal.ppat.1008174)

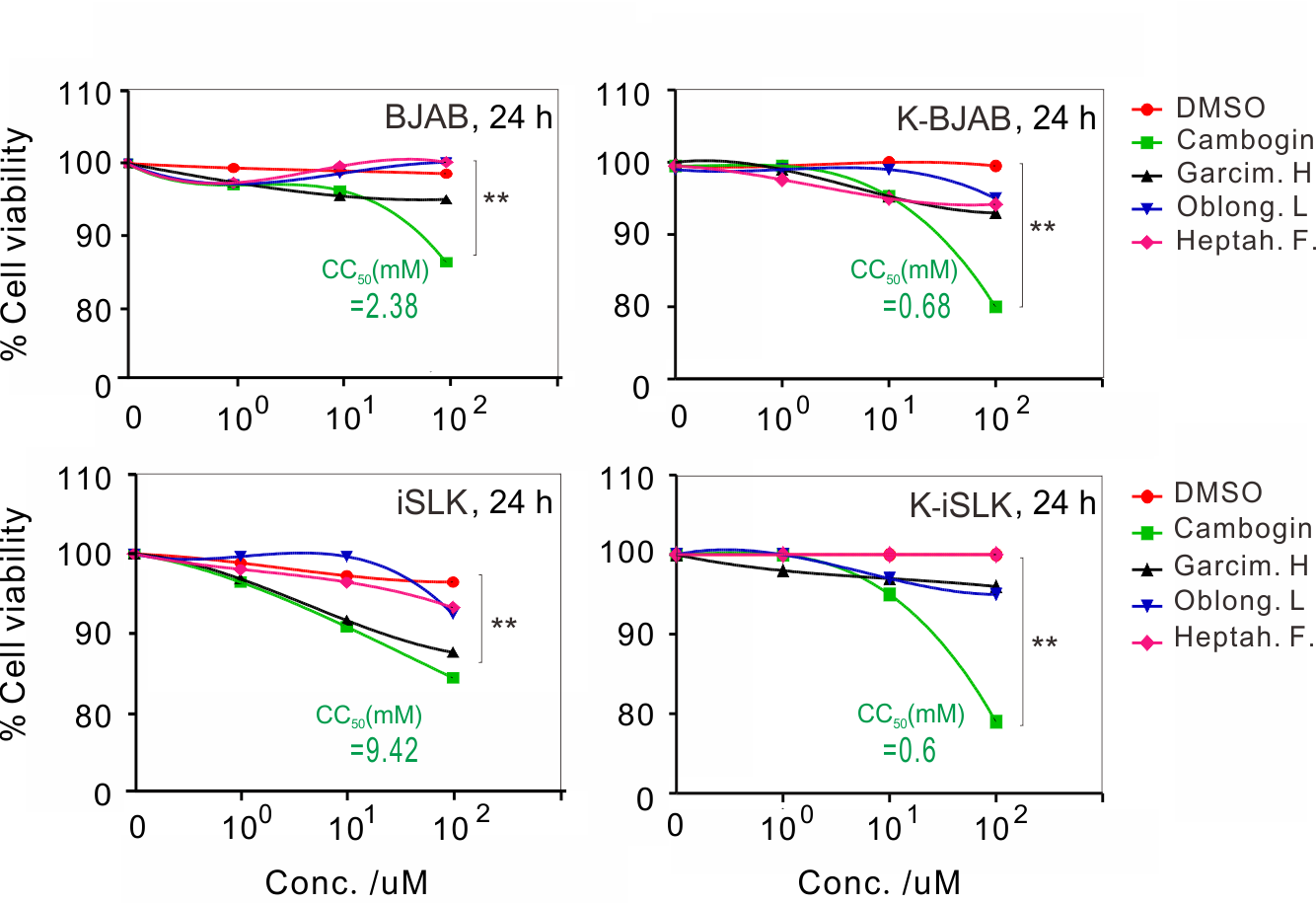

Supplement: S1 Fig — The cell viability of KSHV-infected and uninfected BJAB and iSLK cells treated with different dosage of compounds for 24 h as indicated in figure. The concentration of Cambogin-induced 50% cell death (CC50) was calculated and shown. (TIF) [file ppat.1008174.s002.tif]

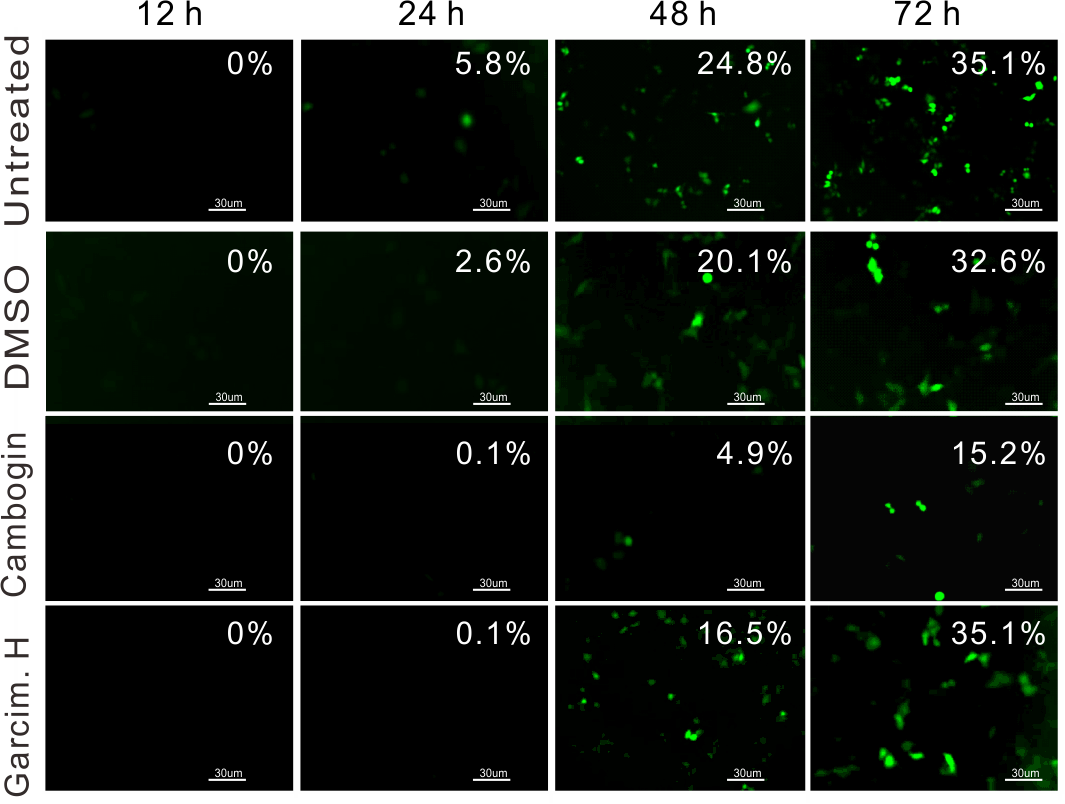

Supplement: S2 Fig — Equal amounts of Hela cells incubated with GFP-tagged KSHV virions (MOI = 2) were untreated or treated with 100 nM Cambogin and Garcimultiflorone H (DMSO as a control) for 12, 24, 48, and 72 h, followed by immunofluorescence analysis to detect the infection efficiency of KSHV. (TIF) [file ppat.1008174.s003.tif]

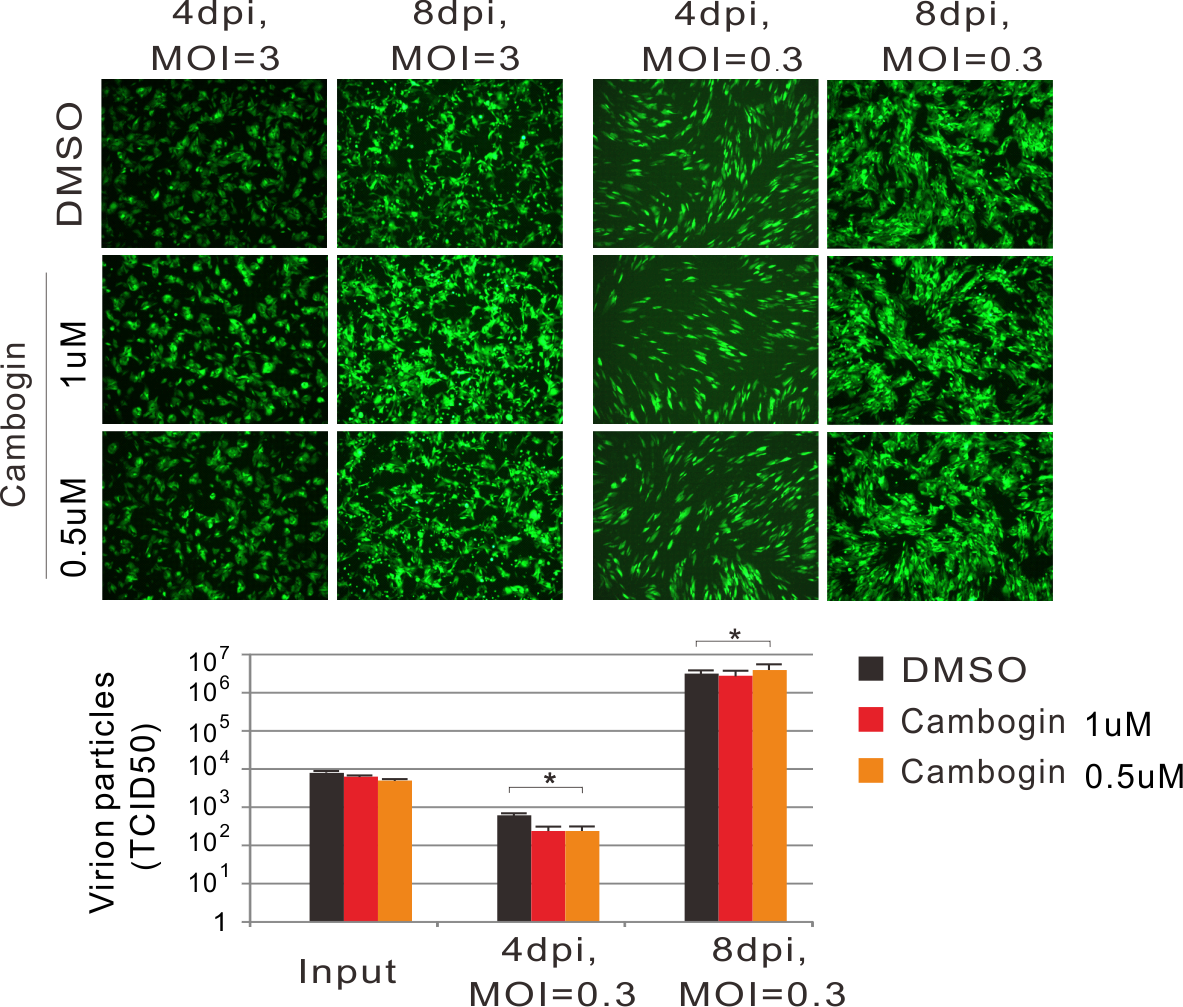

Supplement: S3 Fig — Equal amounts of MRC5 cells incubated with GFP-tagged HCMV (MOI = 0.3 or 3) were subjected to similar treatment and analysis at day 4 or 8 post-infection as described in Fig 5D. The virion particles of TCID50 from supernatants of cells with MOI = 0.3 are shown at the bottom panel. *p > 0.05. (TIF) [file ppat.1008174.s004.tif]

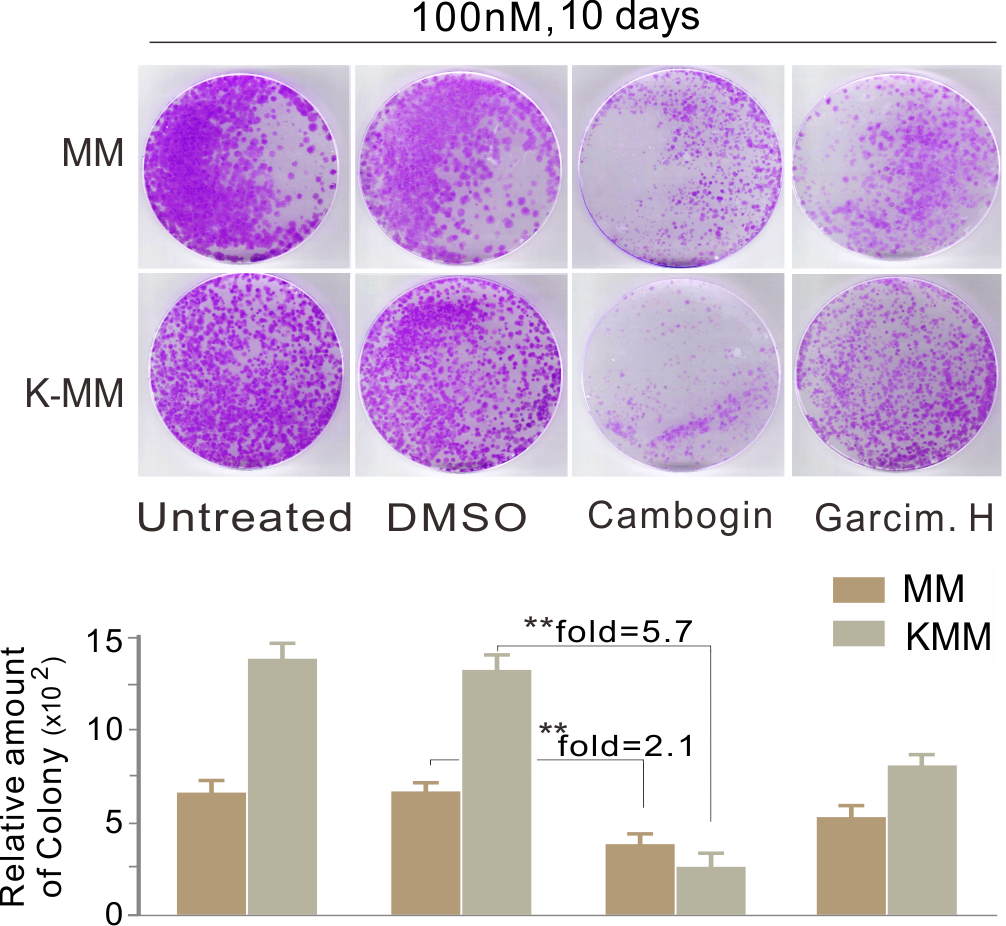

Supplement: S4 Fig — Equal amounts of MM and KSHV-latently infected KMM cells were individually inoculated and treated with or without Cambogin and Garcimultiflorone H and subjected to colony formation assays as indicated. The cells were fixed 10 days later and stained with crystal violet to determine colony number. A representative well with colony formation is shown. Bottom panels, the relative amount of colony formation was calculated from three independent experiments. **p < 0.01. (TIF) [file ppat.1008174.s005.tif]
